# Supplementary material for: WordCommentsAnalyzer: A windows software tool for qualitative research
Source: F1000Res. 2018 Sep 4;7:536. Originally published 2018 May 3. [Version 2] doi: 10.12688/f1000research.14819.2 (PMC6137411; doi:10.12688/f1000research.14819.2)
Supplement: Supplementary file 3 [file f1000research-7-17614-s0002.tgz › b2a79496-4ada-47c4-9682-a99ce867836d.zip!/Eight Iranian Health Journals/Journal of Knowledge and Health_19.docx]

© Journal of Knowledge and Health. This work is licensed under CC BY 4.0 (https://creativecommons.org/licenses/by/4.0/).

Volume & Issue: دوره 8، شماره 3: 1392

Title: اثربخشي نظريه انتخاب به شيوه گروهي بر افزايش تمايز‌يافتگي و صميميت دانشجويان متأهل

http://knh.shmu.ac.ir/index.php/site/article/view/389

مقدمه: ازدواج و انتخاب همسر یکی از مهمترین رویدادهای زندگی انسان محسوب می‌شود که نه تنها بر سلامت جسمی بلکه بر سلامت روانی فرد نيز تأثير مي‌گذارد و به‌طور قطع هر مشکلی برای خانواده اتفاق بیافتد؛ ضرر آن متوجه کل ساختار و نظام ارزشی، فرهنگی جامعه خواهد بود. متأسفانه زوج های جوان وقت و انرژی خاصی صرف آماده شدن برای ورود به این رابطه نمی‌کنند و در همان سال‌های اولیه زندگي، تعارضات قابل توجهی را تجربه می‌کنند. هدف مطالعه حاضر بررسی اثربخشی آموزش نظریه انتخاب بر افزایش تمایز‌یافتگی و صمیمیت زوجین می‌باشد. مواد و روش‌ها: در اين مطالعه مداخله‌اي 30 زوج (60 نفر) از دانشجويان متأهل انتخاب و به‌صورت تصادفي به دو گروه مداخله (15 زوج) و شاهد (15 زوج) تقسيم شدند. قبل از مداخله و بعد از آن پرسشنامه خود متمايزسازي (DSI) و رضايت زناشويي (MIQ) توسط دانشجويان تکميل گرديد. گروه شاهد هيچ آموزشي دريافت نكردند. مداخله شامل 10 جلسه آموزش نظريه انتخاب بود. نتایج: یافته‌ها نشان داد که آموزش نظریه انتخاب به شیوه گروهی بر افزایش تمایزیافتگی دانشجويان متأهل مؤثر است، به‌طوري‌كه ميانگين نمرات خود متمايزسازي در گروه مداخله از 2/19±2/170 به 1/10±8/191 افزايش يافته است. همچنين تأثير مداخله بر افزايش صميميت زناشويي بين زوجين معنادار نبود. نتیجه‌گیری: يافته هاي اين پژوهش سودمندي آموزش نظریه انتخاب را در افزايش تمايزيافتگي تأييد مي‌کند لذا با توجه به تغییرات و تحولات فرهنگی، اجتماعی و نیاز روز افزون جوانان در آستانه ازدواج پیشنهاد می شود در مراکز آموزشی و دانشگاهی، آموزش این مهارت‌ها مورد توجه قرار گیرد.

Title: ارتباط سطوح انسولین با گرلین سرم و هورمون رشد در مردان بزرگسال مبتلا به آسم

http://knh.shmu.ac.ir/index.php/site/article/view/23

مقدمه: جدا از گلوکز، ترشح انسولین به‌وسيله برخی هورمون‌ها و علائم عصبی نیز مهار و یا تحریک مي‌شود. گرلین یک هورمون گوارشی است که ترشح هورمون رشد را تحریک مي‌کند و اخیراً به نقش آن در ترشح انسولین نیز اشاره شده است. هدف از اجرای این مطالعه، تعیین ارتباط بین سطوح پایه انسولین با غلظت‌های گرلین و هورمون رشد در مردان مبتلا به آسم مي‌باشد. مواد و روش‌ها: در این مطالعه، سطوح پایه گرلین و هورمون رشد سرم در مردان چاق بزرگسال مبتلا ‌به بیماری آسم خفیف تا متوسط اندازه‌گيري شد و ارتباط آنها با غلظت انسولین سرم و عملکرد سلول‌های بتای لوزالمعده ارزیابی شد. از آنالیز همبستگی پیرسون جهت تعیین ارتباط انسولین با سایر متغیرها استفاده گردید. نتایج: همبستگی منفی معناداری بین انسولین سرم ناشتا با غلظت‌های گرلین و هورمون رشد مشاهده شد. همچنین ارتباط بین عملکرد سلول‌های بتا با گرلین و هورمون رشد سرم، معکوس و معنادار بود (05/0P<). نتیجه‌گیری: یافته‌های مطالعه حاضر نشان مي‌دهد که افزایش سطوح سیستمیک گرلین و هورمون رشد در بیماران مبتلا به آسم با کاهش ترشح انسولین از پانکراس همراه است و اندازه‌گيري گرلین یا هورمون رشد سرم، یک شاخص پیشگويی‌‌كننده سطوح انسولین خون این بیماران است.

Title: بررسی تأثير جراحی پرولاپس اعضای لگنی بر عملکرد جنسی

http://knh.shmu.ac.ir/index.php/site/article/view/27

مقدمه: اختلال عملکرد جنسی، یکی از مشکلات شایع در زنان مبتلا به پرولاپس اعضای لگنی است. هدف از این مطالعه، ارزیابی عملکرد جنسی در زنان تحت عمل پرولاپس اعضای لگنی مي‌باشد. مواد و روش‌ها: مطالعه حاضر از نوع قبل- بعد است. 50 نفر از زنان مبتلا به پرولاپس اعضای لگنی واجد شرایط ورود به مطالعه، جهت ارزیابی عملکرد جنسی، قبل و بعد از جراحی بررسی گردیدند. ابزار جمع‌آوری داده‌ها شامل مشخصات دموگرافیک، سابقه مامایی و پرسشنامه عملکرد جنسی (Female Sexual Function Index) بود. تجزیه‌و‌تحلیل داده‌ها با استفاده از آزمون‌های آماری کای- دو، t زوجی و رگرسیون خطی چندگانه انجام گردید. نتایج: اختلاف آماری معناداری بین میانگین (±انحراف معیار) عملکرد جنسی براساس FSFI قبل (10/8±19) و بعد (5/2±23/8) از جراحی وجود داشت (0/01=P). همچنین، بهبود معناداری از‌نظر حیطه‌های میل جنسی، تحریک، مرطوب‌شدن واژن، ارگاسم، رضایت و درد بعد از عمل مشاهده گردید (P<0/05). نتیجه‌گیری: عملکرد جنسی در زنان بعد از جراحی پرولاپس اعضای لگنی، بهبود قابل‌توجهی مي‌یابد. لذا انجام مشاوره‌های لازم دراین‌زمینه توصیه مي‌گردد.

Title: بررسي تأثير آموزش مبتني‌بر تئوری رفتار برنامه‌ریزی‌شده بر عملکرد ايمن کارگران

http://knh.shmu.ac.ir/index.php/site/article/view/24

مقدمه: سازمان‌های بهداشتی، رفتار را به‌عنوان عاملي کليدي زيربناي بيماري‌هاي شايع و مشکلات بهداشتي امروزه مي‌دانند. این مطالعه با هدف بررسي  تأثير مداخله آموزشی مبتنی‌بر تئوری رفتار برنامه‌ریزی‌شده در بهبود عملکرد ایمن کارگران صنایع چینی شهرک صنعتی قزوین در نیمه اول سال 1389 طراحی و اجرا شده است. مواد و روش‌ها: تعداد 75 کارگر در هریک از گروه‌های مداخله و شاهد، در یک کارآزمایی نیمه‌تجربی تصادفی کنترل‌شده شرکت کردند. اطلاعات دموگرافيک و داده‌های مرتبط سازه‌های تئوری رفتار برنامه‌ریزی‌شده، آگاهی و عملکرد ایمن در آغاز، 3 ماه و 6 ماه بعد از مداخله، از طريق پرسشنامه خودگزارشي و چک‌لیست عملکرد ایمن جمع‌آوري شد. پانل خبرگان، ضریب آلفای کرونباخ، ضریب آزمون باز آزمون، ضریب کاپا برای تأیید ویژگی‌های روانسنجی ابزار به‌کار رفتند. مداخله آموزشی به همراه کتابچه در قالب 4 کلاس آموزشی 45-60 دقیقه‌ای با حضور 8- 15 کارگر در هر کلاس، به شیوه بحث گروهی با تمرکز بر سازه‌های تئوری رفتار برنامه‌ریزی‌شده اجرا شد. داده‌ها با استفاده از آزمون آناليز واريانس داده‌هاي تكراري تجزیه‌و‌تحلیل شد. نتايج: میانگین سنی شرکت‌کنندگان 7/4‌±31/0 سال بود و 65% آنها مرد بودند. قبل از آموزش، بین دو گروه از حیث متغیرهای دموگرافیک و متغیرهای اصلی پژوهش اختلاف معناداری وجود نداشت. اما پس از آموزش، یافته‌ها نشان‌دهنده تغییر معنادار میانگین امتیاز نگرش (از 5/4±17/2 به 4/4±25/6)، هنجار انتزاعی (از 2/1 ± 4/7 به 1/5±7/0)، کنترل رفتاری درک‌شده (از 1/1± 2/7 به 1/6±6/8)، قصد (از 3/6±8/0 به 2/8±10/9)، رفتار خودگزارشی (از 1/0±2/2 به 1/2± 3/6)، آگاهی (از 1/8 ± 7/0 به 1/5±9/8) و عملکرد ایمن (از 1/4‌±6/3 به 2/1‌±‌8/5‌)، پس از آموزش در گروه مداخله و عدم‌تغییر معنادار متغیرهای مذکور در گروه شاهد بود. نتيجه‌گيري: نتایج تحقیق نشان می‌دهد که مداخله آموزشی بر مبنای سازه‌های تئوری رفتار برنامه‌ریزی‌شده قادر است آگاهی، نگرش، هنجار انتزاعی و کنترل رفتاری درک‌شده کارگران را درباره رفتارهای ناایمن تغییر دهد و موجب بهبود عملکرد ایمنی آنها شود.

Title: رابطه سخت‌رويي با سبك‌هاي مقابله با استرس در دانشجويان

http://knh.shmu.ac.ir/index.php/site/article/view/7

مقدمه: سخت‌رویی، ویژگی شخصیتی است که مقابله مؤثر با استرس را تسهیل کرده، مانع بروز مشکلات جسمی و روانی می‌شود. هدف از این پژوهش، بررسی رابطه سرسختی با سبک‌های مقابله با استرس می‌باشد. مواد و روش‌ها: جامعه آماری در این تحقیق، دانشجویان دانشگاه پیام‌نور گلپایگان بود. از‌طریق نمونه‌گیری خوشه‌ای تصادفي 8 کلاس علوم انسانی و علوم پایه دانشگاه پيام‌نور گلپايگان انتخاب و با مراجعه به این کلاس‌ها 280 دانشجو برگزيده شدند. ابزار جمع‌آوری اطلاعات، پرسشنامه سخت‌رويي اهواز و سبک‌هاي مقابله با استرس جوانان بود. تحليل داده‌ها ازطريق آزمون تفاوت دو میانگین مستقل، تحلیل واریانس و تحليل رگرسيون، گام‌به‌گام انجام شد. نتايج: نتايج، بيانگر عدم‌تفاوت بين دختران و پسران و دو گروه علوم انساني و علوم پايه در ميزان سخت‌رويي بود. تفاوت معناداري در سه سبک مقابله در گروه علوم انساني و علوم پايه وجود نداشت، اما در دو راهبرد رجوع به ديگران و راهبرد مقابله غيرمؤثر، ميانگين زنان بالاتر از مردان بود. بين متغير سخت‌رويي و سبک‌هاي مقابله حل مسئله و رجوع به ديگران، همبستگي معناداري مشاهده شد (001/0>P). نتايج تحليل رگرسيون گام‌به‌گام نشان داد که سبک‌هاي مقابله حل مسئله و راهبرد مقابله غيرمؤثر به‌ترتيب 44% و 47% واريانس سخت‌رويي را پيش‌بيني مي‌کنند. به‌عبارت‌ديگر، رابطه بين سخت‌رويي و سبک‌هاي مقابله با استرس فقط تحت تأثير سبک مقابله حل مسئله و مقابله غير‌مؤثر قرار دارد. نتيجه‌گيري: باتوجه به نتایج حاصل از این تحقیق مبنی‌بر وجود رابطه بین سرسختی و مقابله با استرس می‌توان نتیجه گرفت که ويژگي سرسختي، نگرش دروني خاصي را به‌وجود مي‌آورد که شيوه رؤيارويي افراد با مسائل مختلف زندگي را تحت تأثير قرار مي‌دهد و باعث مي‌شود که فرد فشارهاي رواني را به‌صورت واقع‌بينانه و با بلندنظري مورد توجه قرار دهد. بنابراين تدوين برنامه‌هاي اصلاحي و آموزشي در دانشگاه‌ها مي‌تواند بسيار کمک‌کننده باشد.

Title: بررسي کیفیت زندگی بیماران همودیالیزی

http://knh.shmu.ac.ir/index.php/site/article/view/22

مقدمه: بیماران مبتلا به نارسایی مزمن و پیشرفته کلیه و تحت درمان با همودیالیز، به‌علت درمان‌های دارویی متعدد، مشکلات گوناگون و تغییرات ریشه‌ای در الگوی زندگی خود دارند که بر عملکرد اجتماعی و روانی آنها تأثير می‌گذارد. این مطالعه با هدف تعیین کیفیت زندگی بیماران همودیالیزی شهرستان‌های آبادان و خرمشهر در سال 1390 انجام شده است. مواد و روش‌ها: اين پژوهـش مطالعه‌اي مقطعی از نوع توصيفـي تحلیلی است. جامعه مورد مطالعه شامل تمامی افرادی بود که به‌صورت مستمر به کلینیک‌های همودیالیز شهرهای آبادان و خرمشهر )80 نفر( مراجعه می‌کردند و با استفاده از پرسشنامه‌ای كه از دو بخش اطلاعات دموگرافيك و پرسشنامه کیفیت زندگی بیماران کلیوی(KDQOL-SF)  تشكيل شده بود، مورد بررسی قرار گرفتند. تجزيه‌و‌‌‌‌تحليل داده‌ها با استفاده از آزمون‌هاي آماري آناليز واريانس، همبستگي پيرسون و آزمون t مستقل انجام شد. نتايج: ميانگين نمره كيفيت زندگي نمونه‌ها در این پژوهش 82/8 ± 22/47 و بيانگر كيفيت زندگي پایین آنها مي‌باشد. یافته‌های اين پژوهش نشان داد كه بين متغير كيفيت زندگي و سن (5/0=r، 04/0=P) و کیفیت زندگی با مدت ابتلا به نارسايي مزمن كليه (03/0=P، 23/0=r) ازنظر آماری رابطه معناداری وجود دارد. ارتباط مثبت معناداري بين تحصيلات و كيفيت زندگي (003/0=P) و نيز بين میزان درآمد افراد و کیفیت زندگي (0001/0P<) مشاهده شد.  نتیجه‌گیری: کیفیت زندگی بیماران تحت درمان با همودیالیز پایین بوده و نیازمند توجه بیشتر مسئولان به حمایت‌های اجتماعی و رسیدگی به وضعیت رفاهی این افراد است.

Title: بررسی اثرات سولفات منیزیم و ورزش منظم روی آستانه درد موش‌های دیابتی

http://knh.shmu.ac.ir/index.php/site/article/view/33

مقدمه: نوروپاتی محیطی، یکی از عوارض مهم دیابت ملیتوس می‌باشد. ازطرفی، نشان داده شده است که هیپرگلیسمی و استرس اکسیداتیو از عوامل مهم در ایجاد و پیشرفت نوروپاتی در دیابت می‌باشد. باتوجه به اینکه تمرینات ورزشی منظم و مصرف سولفات منیزیم  قادر به کاهش قند خون ناشی از دیابت بوده و اثرات ضد اکسیداتیو دارند، در این مطالعه اثرات آنها روی آستانه درد موش‌های دیابتی بررسی شده است. مواد و روش‌ها: در این تحقیق، موش‌های صحرایی نر نژاد ویستار به تعداد 30 سر در محدوده وزنی 10±220 گرم با تزریق زیرجلدی داروی استرپتوزوسین (60 میلی‌گرم/ کیلوگرم) دیابتی شدند. یک هفته بعد از القای دیابت، حیوانات به مدت 8 هفته تحت تمرینات منظم ورزشی شنا و مصرف سولفات منیزیم قرار گرفتند. به این ترتیب که حیوانات ابتدا 60 دقیقه به‌مدت 3 هفته و سپس 90 دقیقه به‌مدت 2 هفته و درنهایت 120 دقیقه به‌مدت 3 هفته، تحت تمرین شنا در آب 32 درجه قرار گرفتند. سولفات منیزیم (10 گرم/لیتر) به‌مدت 8 هفته به آب خوراکی حیوانات اضافه گردید. 48 ساعت بعد از پایان هر مرحله، از حیوانات تست Tail- Flick جهت اندازه‌گیری آستانه درد به‌عمل آمد. مقايسه ميانگين‌ها با استفاده از آزمون t مستقل و آناليز واريانس داده‌هاي تكراري انجام شده است. نتایج: دیابت به‌مدت 5 و 8 هفته به‌طور معناداری آستانه درد موش‌ها را کاهش داده است (01/0>P). تمرینات منظم ورزشی و مصرف سولفات منیزیم، هیپرآلژزی ناشی از دیابت را به‌طور معناداری کاهش دادند (05/0>P). همچنين اثرات توأم تمرینات منظم ورزشی و مصرف سولفات منیزیم در کاهش آستانه درد بیشتر از اثر هریک آنها به‌تنهایی می‌باشد (01/0>P). نتیجه‌گیری: تجویز تمرینات منظم ورزشی و سولفات منیزیم در دیابت مي‌تواند روشی مناسب برای پیشگیری و برطرف‌کردن هیپرآلژزی ناشی از آن باشد. درنتیجه با انجام مطالعات بیشتر در آینده شاید برای درمان و کنترل درد مفید مي‌باشند.

Title: برآورد ظرفیت هوازی و ظرفیت کار فیزیکی در کارگران

http://knh.shmu.ac.ir/index.php/site/article/view/34

مقدمه: اندازه‌گیری حداکثر ظرفیت هوازی، اهمیت زیادی در ایجاد تناسب و تطابق فیزیولوژیک بین کارگر و کار دارد. این مطالعه با هدف برآورد ظرفیت هوازی و ظرفیت کار فیزیکی کارگران بخش گالوانیزه شرکت نورد لوله سمنان و نیز تعیین فراوانی نسبی کارگران دارای شغل متناسب با ظرفیت کار فیزیکی انجام شده است. مواد و روش‌ها: در این مطالعه مقطعی، 50 نفر از کارگران مرد بخش گالوانیزه شرکت نورد لوله سمنان به‌روش تصادفی مورد بررسی قرار گرفتند. از روش شاهنواز و تاکسورت (tuxworth & shahnavaz) برای اندازه‌گیری حداکثر ظرفیت هوازی این افراد استفاده شد. داده‌هاي به‌دست‌آمده پس از ورود به نرم‌افزارSPSS  به كمك آزمون‌هاي آماري t مستقل و ضريب همبستگي تجزيه‌و‌تحليل شدند. نتايج: میانگین حداکثر ظرفیت هوازی کارگران مورد مطالعه 33/0±88/2 لیتر در دقیقه و میانگین ظرفیت کار فیزیکی آنها 54/0±76/4 کیلوکالری بر دقیقه برآورد گردید. ارتباط معناداری بین ظرفیت هوازی با شاخص توده بدن و بین ظرفیت هوازی (برحسب میلی‌لیتر برکیلوگرم بر دقیقه) با سن وجود داشت. نتایج نشان داد که 36% از كارگران در طول شیفت ‌کاری برای انجام وظایف شغلی، انرژی بیشتری از ظرفیت کار فیزیکی خود مصرف می‌کردند. نتیجه‌گیری: باتوجه به طبقه‌بندی سازمان بین‌المللی کار، میانگین ظرفیت کار فیزیکی کارگران مورد مطالعه، در رده میزان انرژی مصرفی در کار سبک قرار می‌گیرد. بنابراین به‌طور متوسط این کارگران دارای توانایی جسمانی انجام کارهای سبک یا کمتر هستند. بیش از یک‌سوم این کارگران نیز نیازمند تعدیل وظایف شغلی با تغییر شغل خود به شغلی با مصرف انرژی کمتر هستند.

Title: صنعت داروسازي در عصر صفوي

http://knh.shmu.ac.ir/index.php/site/article/view/35

مقدمه: باتوجه به سابقه چند هزار ساله‌ طب سنتي ايران و شاخه‌هاي متعدد آن، ازجمله داروسازي، و اختصاص كتب و رسالات فراوان برجاي‌مانده از قدما در اين حوزه، اين پژوهش در پي يافتن جايگاه صنعت داروسازي در عصر صفوي مي‌باشد. مواد و روش‌ها: اطلاعات و داده‌هاي اين تحقيق مبتني‌بر تفحص در كتب و رسالات پزشكي و داروسازي دانشمندان ايراني در عصر صفويه و اعصار ماقبل آن مي‌باشد. اين اطلاعات از منابع مذكور گردآوري، تجزيه‌وتحليل و سپس براي تبيين اين بحث به‌كار گرفته شده است. نتايج: براساس بررسي‌هاي انجام‌يافته مشخص گرديد كه گرچه عصر صفويه در صنعت داروسازي و طب سنتي ايران از جايگاه خوبي نسبت به ديگر كشورها برخوردار بوده، هيچ‌گاه پزشكاني همانند محمدبن‌زكرياي رازي، ابن سينا، علي‌بن‌ربن طبري، سيداسماعيل جرجاني و ... و آثار گران‌سنگي چون الحاوي، القانون في‌الطب، ذخيره خوارزمشاهي و ... در اين دوره به‌وجود نيامد. نتيجه‌گيري: براساس مطالعات انجام‌شده در حوزه صنعت داروسازي، مشخص گرديد كه علي‌رغم فراز و نشيب‌هاي تاريخي، ايرانيان هميشه در داروسازي و شناخت گياهان دارويي سرآمد اقران خويش بوده‌اند. باتوجه به تنوع اقليمي و رويش بسياري از گياهان دارويي در ايران و يا امكان كشت آنها و همچنين دردست‌بودن كتب و رسالات قدما دراين‌زمينه مي‌توان در جهت احياي اين صنعت مادر، اقدامات مفيدي انجام داد. اين كار مي‌تواند به احياي طب سنتي ايران كمك شاياني نموده و ميراث چندين هزارساله ما را از فراموشي و نابودشدن مطلق نجات دهد.
